# Primary renal lymphoma—a case report

**DOI:** 10.3332/ecancer.2014.466

**Published:** 2014-09-25

**Authors:** N Geetha, Abdul Shahid, Varun Rajan, Priya Mary Jacob

**Affiliations:** 1 Department of Medical Oncology, Regional Cancer Centre, Trivandrum 695011, India; 2 Department of Pathology, Regional Cancer Centre, Trivandrum 695011, India

**Keywords:** NHL, Kidney

## Abstract

Primary renal lymphoma is a rare entity representing less than 1% of lesions in the kidney. We present the case of a 42-year-old male who was evaluated for pain and a mass in the abdomen. The computed tomogram of the abdomen showed a large lobulated homogeneously enhancing mass lesion of about 14×12×18 cm, involving the whole of the left kidney and encasing the left renal vessels and ureter. The patient underwent a biopsy, and the histopathology was diffuse large B cell lymphoma, positive for LCA, CD20, PAX 5, Bcl 2 and negative for SIgM, CD33, CD34, CD5, Tdt, with MIB 1 labelling index of 40%. He received chemotherapy with rituximab, cyclophosphamide, doxorubicin, vincristine and prednisolone (R CHOP) for eight cycles followed by radiation to the residual mass and achieved complete remission. Currently, he is alive and in remission at 28 months.

## Introduction

Primary renal lymphoma (PRL) is a rare entity of the genitourinary tract in which the disease is localized in the kidneys or in which renal involvement is the presenting feature [1]. Renal involvement is detected in 3–8% of all patients undergoing staging for non-Hodgkin’s lymphoma (NHL); however, in autopsy series, renal involvement has been reported in 30–60% [2]. PRL represents less than 1% of the lesions in this organ [3]. Here, we report on a patient with PRL.

## Case Report

**Figure 1.**
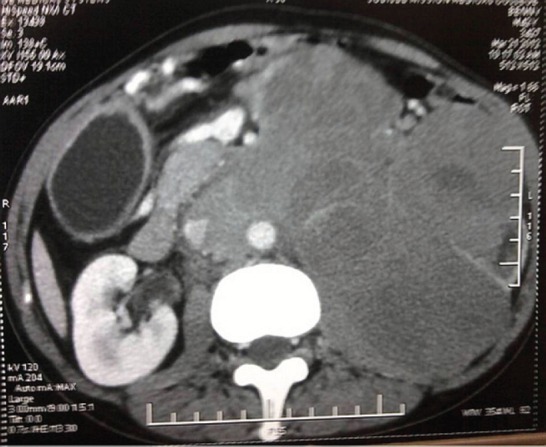
A contrast-enhanced CT of the abdomen showing large, lobulated homogenously enhancing mass lesion 14×12×18 cm arising from left kidney and encasing the renal vessels.

**Figure 2.**
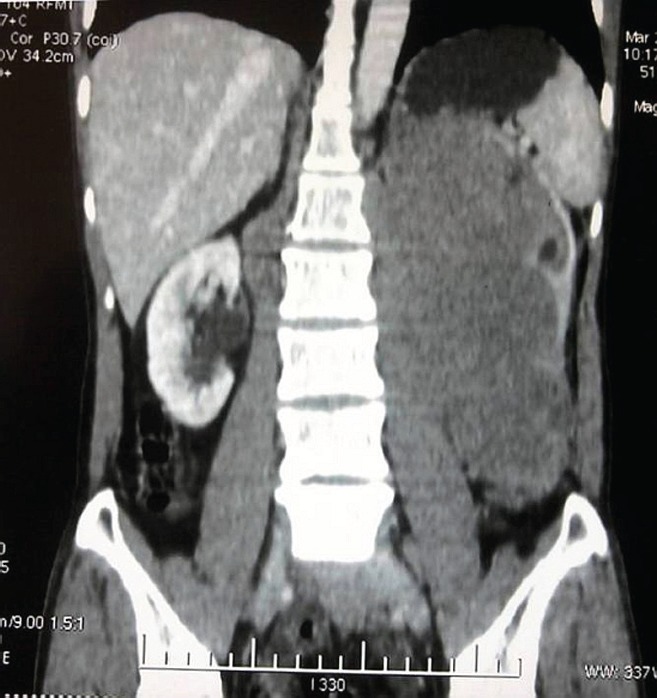
A coronal view showing the mass encasing the left ureter, left renal artery, and vein. The right kidney appears normal.

**Figure 3.**
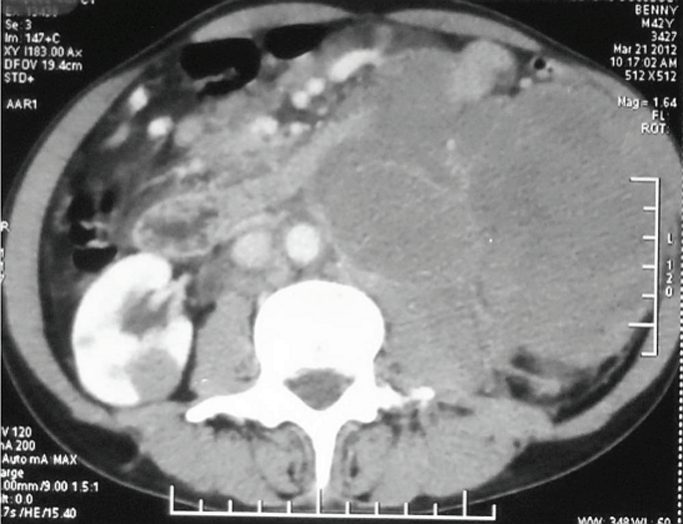
A urographic sequence of the left kidney does not show any excretion.

A 42-year-old male was evaluated for abdominal pain and a mass in the abdomen at the local hospital. The examination showed a firm lobulated mass on the left side of the abdomen. The ultrasonogram showed a large renal mass on the left side, crossing the midline anterior to the aorta, extending to the epigastrium, left iliac region and involving the tail of the pancreas. The computed tomogram (CT) of the abdomen with urographic sequence showed a large lobulated homogeneously enhancing mass lesion of about 14×12×18 cm, involving the whole of the left kidney and encasing the left renal vessels and ureter (Figures 1 and 2). The mass was seen to be displacing the bowel loops to the right side and the splenic vessels anteriorly. On urographic sequence, the left kidney did not show any excretion (Figure 3). Multiple enlarged lymphnodes in the paraaortic, retrocaval, renal hilar, mesenteric region of 10–40 mm in size were present.

**Figure 4.**
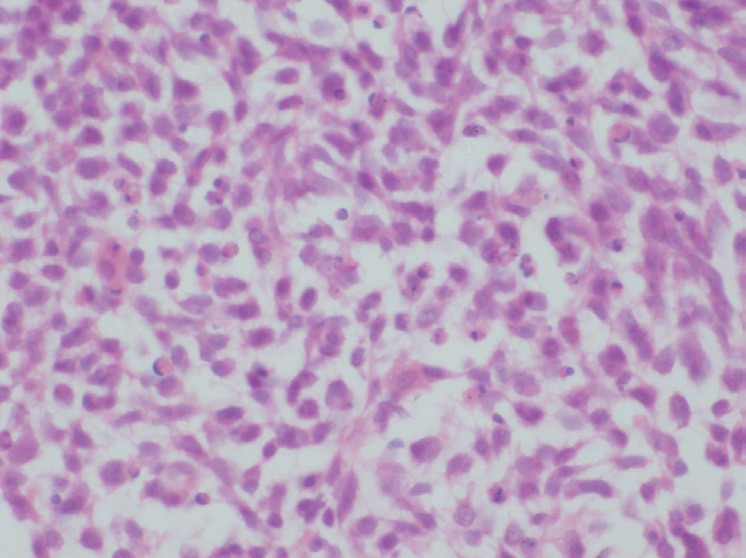
H & E × 400: Tumour cells have moderate cytoplasm, large vesicular nuclei.

**Figure 5.**
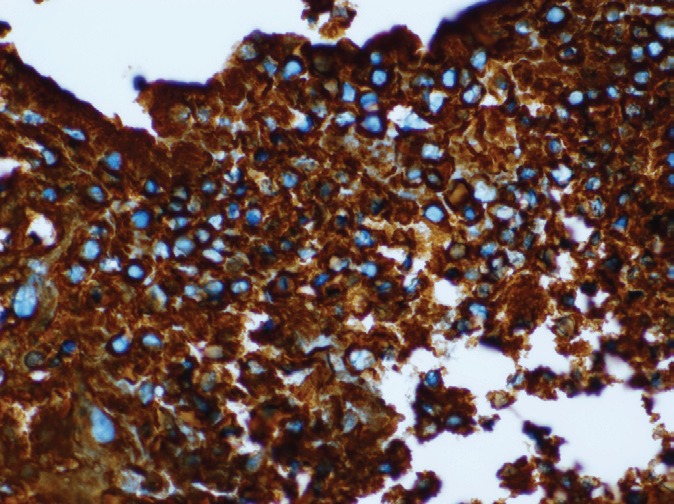
IHC, CD 20 × 400: Tumour cells are CD 20 positive.

**Figure 6.**
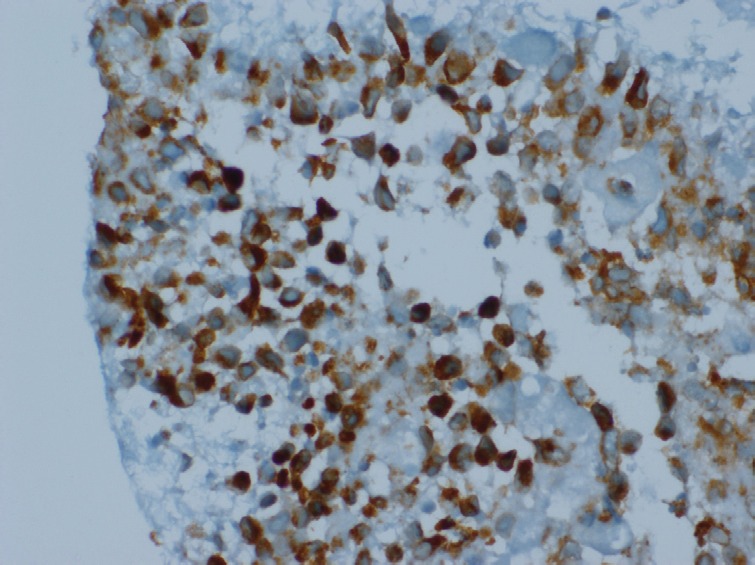
IHC, Bcl 2 × 400: Tumour cells are Bcl 2 positive.

The patient underwent biopsy of the mass and subsequently presented to us. He did not have B symptoms. There were no palpable lymphnodes or hepatosplenomegaly or any other mass. His haemogram was normal, and serum chemistries were normal except for a serum creatinine of 1.7 mg/dl and LDH of 2320 U/dl. The histopathological examination of the biopsy showed predominantly large cells with moderate cytoplasm, large vesicular nuclei, prominent nucleoli (Figure 4). Immunohistochemistry analysis showed that the tumour cells were positive for LCA, CD20, PAX 5, Bcl 2 and negative for SIgM, CD33, CD34, CD5, Tdt and MIB 1 labelling index was 40% (Figures 5 and 6). The diagnosis was NHL diffuse large B cell type (DLBCL).

The CT of chest and the biopsy of bone marrow were normal. He received chemotherapy with rituximab, cyclophosphamide, vincristine, doxorubicin, and prednisolone for eight cycles (RCHOP). A positron emission tomogram at the end of chemotherapy showed the perinephric region to be FDG avid, and he was consolidated with irradiation of 36GY/18#. A repeat CT showed no evidence of disease. He is alive in complete remission at 28 months.

## Discussion

PRL is defined as lymphoma arising in the renal parenchyma and not resulting from the invasion of an adjacent lymphomatous mass [4]. Secondary involvement of the kidneys by lymphoma usually occurs in disseminated cases. PRL comprises only 0.7% of extranodal lymphomas [5]. Renal involvement is more common in the NHL, and mostly, they are intermediate or high grade B cell lymphomas [5]. PRL is a rare and debated entity since the kidneys are devoid of lymphatic tissue [6]. The proposed pathogenetic mechanisms include origin in the lymphatics of renal capsule or subcapsular lymphatic tissue, with subsequent invasion of the renal parenchyma or any chronic inflammatory process might be the cause of lymphomas [7].

It usually occurs in the middle aged, with the presenting symptoms being flank pain and abdominal mass. The laboratory values are usually normal except for an elevated serum creatinine [4]. Renal lymphoma may present as a solitary mass (10–20%) or multiple mass (60%), and extend by contiguity (25–30%), diffuse infiltration (20%) or perirenal involvement (10%) [8]. Our patient also had a similar presentation.

Yasunaga *et al* reported eight cases of PRL, six being DLBCL, and none had lymphadenopathy or hepatosplenomegaly at presentation. Their mean survival was six months. In autopsy, only one was limited to the kidney, and the others had extrarenal visceral invasion [7]. A patient with a solitary renal mass invading Gerota’s fascia, with lymph nodes in the mediastinum and retroperitoneum was reported by Pinggera *et al*, and this case was accepted as possible PRL [6]. Three cases with solitary mass, each one with paraaortic, retrocaval conglomerated lymph nodes and invasion of renal vein and perirenal adipose tissue were described [9]. All cases were accepted as PRL, NHL DLBCL. A 62-year-old male with PRL with lung nodules who had a nephrectomy done and histology was DLBCL [2]. Our patient also had NHL DLBCL positive for LCA, CD 20, PAX5 and BCL 2. A 23-year-old woman with primary renal DLBCL received RCHOP and is alive at 15 months [10]. Pahwa reported on a 39-year-old male with left PRL with local invasion who underwent radical nephrectomy. He was treated with CHOP and was alive at two years [11]. Vedovo *et al* described an 82-year-old female who underwent radical nephrectomy for a recurrent renal mass and was diagnosed as lymphoplasmacytic MALT lymphoma [12].

In short, there has been no concordance between researchers. Some studies empahsise the absence of lymph node involvement and leukaemic blood picture to make a PRL diagnosis, but in other studies, concurrent lymph node involvement alongside a solitary renal mass, is accepted PRL [7]. Our patient also had a few small paraaortic lymph nodes in addition to the huge renal mass.

It is a diagnostic challenge to differentiate renal lymphoma from other renal masses, especially in cases of unilateral lesions since they simulate renal carcinomas both radiologically and pathologically. Renal lesions that lack typical radiological features of renal cell carcinoma or multi-nodular involvement of the kidney along with lymphadenopathy merits a biopsy. A solitary unilateral renal mass, perirenal mass with distortion of the renal architecture in the CT and the absence of lymph node enlargements are more suggestive of renal cell carcinomas [13]. The standard management of a renal mass is nephrectomy. However, primary renal lymphoma is an exception in which patient can be treated with systemic chemotherapy. Therefore, in spite of its uncommon occurrence, it is of immense importance to distinguish primary renal lymphoma from renal carcinoma.

In view of its aggressive nature and poor prognosis, it is important to make an early diagnosis and start treatment promptly. The treatment of choice is systemic chemotherapy using a CHOP regimen. The earlier reviews report a poor prognosis for patients with PRL, with a median survival of less than one year [4, 7], but the recent reports suggest a better survival probably due to the addition of rituximab to the combination chemotherapy [9, 11]. The present case also received RCHOP and is alive in remission after two years.

Although PRL is a rare tumour type, it must be taken into account when making a differential diagnosis of any renal mass. A high index of suspicion for PRL should be kept if the lesion shows multi-nodular involvement of the kidney along with lymphadenopathy. An effort should be made to make a preoperative diagnosis since primary renal lymphoma can be treated with systemic chemotherapy unlike other renal tumours where one requires nephrectomy.

## Conflicts of interest

The authors have no conflicts of interest to declare.
